# Examining the wider context of formal youth mentoring programme development, delivery and maintenance: A qualitative study with mentoring managers and experts in the United Kingdom

**DOI:** 10.1016/j.childyouth.2018.10.028

**Published:** 2018-12

**Authors:** Heide Busse, Rona Campbell, Ruth Kipping

**Affiliations:** Bristol Medical School, Population Health Sciences, University of Bristol, Bristol, United Kingdom

**Keywords:** Mentoring program, Adolescent, Context, Qualitative research, United Kingdom, Expert (E), Manager (M), United Kingdom (UK), United States of America (USA)

## Abstract

Mentoring programmes are commonplace, yet little is known about the circumstances in which they operate. This study aimed to gain insight into the context surrounding youth mentoring programmes by asking programme managers and experts in the United Kingdom about their experiences. Telephone interviews with twenty-three programme managers and five experts were undertaken. Interviews were recorded, transcribed verbatim and analysed iteratively using thematic analysis. Contextual influences at the individual-, interpersonal-, organisational-, community-, policy-, and societal-level were identified to impact on programme's development, delivery and maintenance and were summarised in a model. This study further found that youth mentoring programmes operate within a complex context. This context provides challenges and opportunities that impact on programme's sustainability; resulting in many externally-funded programmes to ‘hang by a thread’. It is important for service providers, commissioners and academics to recognise the complexity surrounding mentoring programmes to ensure that programmes are delivered as intended and evaluated appropriately.

## Highlights

• Formal mentoring programmes in the United Kingdom operate within a complex context
• Individual-, interpersonal-, organisational-, community-, policy-, and societal-level influences were identified
• A conceptual model was established highlighting the range of contextual influences on formal mentoring programmes
• The context provides challenges and opportunities that impact on programme's sustainability

## 1 Introduction

Mentoring programmes are commonplace in various settings and contexts in developed countries. Previous evaluations have highlighted the potential benefits of such programmes in improving young people's health, social and educational outcome's (DuBois et al., 2011; Eby et al., 2008; Herrera et al., 2011). However, there is limited knowledge about the influence of context on mentoring programmes and programmes' effectiveness and it is uncertain whether findings from past evaluations, predominantly undertaken in the United States of America (USA), are transferrable to other settings and countries (Philip, 2003).

Context can be defined as the “circumstances or events that form the environment within which something exists or takes place” (Poland et al., 2006, p. 59). In other words, context can be seen to include and refer to “anything external to the intervention” (Moore et al., 2015), which might include the social, political and/or organisational setting in which an intervention is developed, delivered or evaluated (Rychetnik et al., 2002b). Understanding the context is particularly important with regard to complex interventions as these interact with the context in which they find themselves in (Bonell et al., 2012; Craig et al., 2008). In fact, contextual factors can influence whether or not an intervention is effective (Bonell et al., 2006; Hawe et al., 2004). For instance, contrary to the positive findings of USA trials (Olds, 2006), an evaluation of an adapted version of the Family-Nurse Partnership intervention found no evidence of effectiveness in the United Kingdom (UK) (Robling et al., 2016) in improving early maternal and child health. The authors argued that this might be because teenage mothers in the UK, unlike the USA, benefit from a well-established health visitor service (Robling et al., 2016), however, others argued that this could equally be due to the outcomes that were selected to investigate the effectiveness of the intervention (Barlow et al., 2016).

Having a thorough understanding of the context surrounding an intervention is therefore crucial for: (i) understanding the intervention's possible reach, efficacy, adoption, implementation and maintenance (Glasgow et al., 1999), (ii) determining the underlying mechanisms and key intervention components by which an intervention is assumed to work or lead to a change and (iii) aiding the judgement about internal and external validity of an intervention (Moore et al., 2015; Rothwell, 2005). Moreover, having a good understanding of the implementation context, the unique social determinants, and the needs and motivations of the target population is critical to the potential success of a public health intervention (Rychetnik et al., 2002a).

Understanding the context of public health interventions is important, yet there is an overall lack of knowledge of the contextual factors at play (Shoveller et al., 2016). A Cochrane review of 67 complex interventions in line with the World Health Organisation's (WHO) health-promoting school framework revealed limited use of intervention theories and an overall lack of contextual and process data (Langford et al., 2014). Additionally, few public health trials make a formal assessment of the generalisability and replicability of their findings (Bonell et al., 2006). Widely used guidelines for the reporting for interventions, such as the CONsolidated Standards Of Reporting Trials (CONSORT) statement (Schulz et al., 2010), the RE-AIM framework (Glasgow et al., 1999) and the Template for Intervention Description and Replication (TIDieR) checklist (Hoffmann et al., 2014) provide vague guidelines about what contextual information should be reported. The Oxford Implementation Index, a tool to capture implementation data, recommends that information should be provided on the study's setting, geographical location and date/time, information about participant characteristics, characteristics of the delivering organisations and service environment, the unique ethical considerations and reporting of the external events occurring at the same time of the intervention in order to obtain insight into the context within which interventions were implemented (Montgomery et al., 2013). More recently, the first specific guidelines for the appraisal of context for complex public health interventions have been published. These include the Context and Implementation of Complex Interventions (CICI) framework published in 2016 (Pfadenhauer et al., 2017) and guidance published by the Canadian Institute of Health Research Context Guidance authors group (Craig et al., 2018).

In sum, knowledge about how interventions operate in different contexts and the extent to which contextual factors can impact on the evaluation of a given intervention is incomplete, which might partly be explained by the limited reporting of context required by existing guidelines.

Contextual factors in youth mentoring programmes have also been neglected so far in most previous evaluations (DuBois et al., 2011). This is despite the fact that the most commonly used model of youth mentoring in the literature (Rhodes, 2005; Rhodes et al., 2006) explicitly details that programme practices and the wider family and community context can impact on the programme outcomes. Further, various programme characteristics have been identified as critical to the success of a programme (Rhodes and Dubois, 2008). For instance, the duration of mentoring (DuBois and Silverthorn, 2005; Grossman and Rhodes, 2002), type of mentor (DuBois and Silverthorn, 2005) and the mentee-mentor relationship characteristics (DuBois and Silverthorn, 2005; Langhout et al., 2004) are all factors that have been associated with differential outcomes in mentoring.

Only a handful of studies have specifically focussed on contextual influences within youth mentoring programmes, only one of which was within the evaluation of mentoring programmes (Farruggia et al., 2011). For example, based on an analysis of essays written by mentors of elementary school children in the United States, Pryce and colleagues (Pryce et al., 2015) explored the experience of mentors with mentees' peers in the context of a site-based mentoring programme. Whereas mentee's peers do not usually feature in formal mentoring programmes, authors found that peers of the mentee can interact in different ways with the mentoring relationship. This led authors to emphasise the importance of looking beyond the dyad between mentor and mentee to looking at mentoring as a system (Pryce et al., 2015). Lakind et al. (2015) interviewed professional mentors working with youth at risk for adjustment problems about their mentoring role and their perceptions of their mentees and revealed that mentors perceived the prevalence of their mentees' risks as by-products of environmental adversity they are faced with (Lakind et al., 2015). Thus, mentors reported spending time and effort to work with key individuals in the life of their mentees, highlighting how the mentee's environment was perceived to give rise to challenges and opportunities and can impact on the mentoring relationship (Lakind et al., 2015). In an attempt to examine whether mentoring programmes within New Zealand were culturally-appropriate, Farruggia et al. (2011) undertook a systematic review and concluded that programmes seemed to be less appropriate for minority youth and that cultural-appropriateness seemed to be related to the programme's effectiveness.

Studies that focussed on the context of mentoring programmes are limited in that they were oftentimes focussed on programmes other than youth mentoring programmes (Barnett, 2008; Freedman and Baker, 1995; Holley and Caldwell, 2012; Ssemata et al., 2017) and did not include an investigation of the wider socio-economic, organisational, historical, political or other contextual factors surrounding programmes (Langhout et al., 2004; Pryce et al., 2015). Moreover, whereas previous studies were often undertaken with mentors or mentees, who can give some insight into the challenges and opportunities that are experienced (Brady et al., 2017; Lakind et al., 2015; Pryce et al., 2015; Spencer, 2007; Spencer et al., 2017), there has been a limited focus on the experiences of those who design, develop and manage the mentoring programmes and the context within which they operate.

With recent developments and a rise of mentoring in the UK and other developed countries, assessing the generalisability of USA-based studies and programmes is vital. Developments in mentoring in the UK have been widely influenced by the USA as most evidence comes from there (Hall, 2003; Philip, 2003; Philip and Spratt, 2007). It is not known whether findings are generalisable cross-culturally and what role the wider context plays in mentoring. As Philip noted, “a critical approach needs to be taken to the current reliance by mentoring interventions on North America” (Philip, 2003, p. 111). Unlike the deeper insights into the context of mentoring in Ireland that have been provided (Brady and Curtin, 2012; Brady and Dolan, 2009; Dolan and Brady, 2007), existing evaluations of mentoring programmes in the UK only briefly address the issue of context and as most were conducted around the millennium (Shiner et al., 2004; St James-Roberts et al., 2005; Tarling et al., 2001), it is difficult to judge whether these findings are still relevant. To our knowledge no study has yet explored the wider context or the design, development and management of youth mentoring programmes within the United Kingdom.

We undertook interviews with programme managers of youth mentoring programmes and experts in the field of mentoring in the United Kingdom about their experiences of the development, delivery and maintenance of formal mentoring programmes for young people and the context within which formal mentoring programmes operate.

## 2 Methods

### 2.1 Participants

Programme managers from purposefully sampled mentoring organisations and selected experts in the field were invited to take part in semi-structured telephone interviews. Eligibility criteria for mentoring organisations were that they had to be involved in the delivery of one or more formal mentoring programmes to young people aged 11–16 years that were currently enrolled in secondary schools in the UK. Mentoring in the research was defined as ‘any programme between an identified young person and other individual(s), aimed at the support of the young person’. Therefore, mentoring organisations that provided any type of mentoring, any type of mentor and any type of aims and objectives were eligible for inclusion. An overview of common terms related to mentoring used in this paper is provided in Table 1.

**Table 1:** Overview of common terms used in this paper.

Common terms used in this paper	Description
Mentor	The individual supporting, guiding and helping another, usually more inexperienced, individual. Sometimes referred to as ‘volunteers’.
Mentee	The individual receiving mentoring; also referred to as protégé(e) or apprentice.
Matching	The process of pairing up a mentor with a mentee.
School-based mentoring	Programmes that predominantly take place within the school, either as part of school, before or after school but on school grounds.
Community-based mentoring	Programmes that predominantly take place in the community.
One-to-one mentoring	Programmes in which one mentor is working with one young person and in which matching typically takes place.
Group mentoring	Programmes in which a group of mentors works with a group of young people, or in which multiple mentors work with a single young person or in which multiple young people work with one mentor. These can be school- or community-based.
Peer mentoring	Programmes in which older students mentor younger students. These are typically students within the same school and the mentoring programme takes place within the school setting. Often, these are internally-run schemes by the school monitored by an allocated staff member.
Online mentoring	Programmes in which mentor and mentee communicate via online technology with one another. This might include an initial face-to-face meeting but the mentoring as such is then carried out using the internet.
Mentoring relationship	The relationship between the mentor and mentee; in the case of this study, the relationship which has been created through the mentoring programme.
Mentoring provider	The organisation, institution or team that provides the mentoring programme.
Formal mentoring	Formal mentoring indicates that the relationship between mentor and mentee is formalised and explicitly recognised. In most instances, this means that both mentor and mentee are taking part in a mentoring programme that explicitly recognises the mentoring relationship and usually involves matching a selected young person (mentee) to another individual (mentor).
Exit strategy	The strategy that details how the end of the mentoring relationship is managed.

A thorough search of national mentoring websites, charitable organisations and turst registers were undertaken to identify current organisations and programmes. From a total of 815 organisastions that were identified from the search, 163 met the eligibility criteria for inclusion in the study, namely that they were provided for young people and that that they entailed mentoring programmes rather than other types of intervention programmes such as befriending, coaching or counselling. Maximum variation sampling was used to recruit a purposeful sample of programme managers from within the eligible organisations. Mentoring organisations were purposefully selected by country within the UK and by the type of mentoring provided to ensure that different perspectives and experiences were captured. Participants who took part in the research study were asked whether they knew of other mentoring programmes that met study eligibility criteria. As such, snowball sampling was used in combination with maximum variation sampling to obtain a varied sample of organisations. Programme managers were selected as participants because they were seen to be best placed to talk about the operational aspects of the programme, the programme's historical development as well as challenges, opportunities and concerns experienced at an organisational level. Of the 163 eligible organisations, a total of 29 eligible and varied organisations were contacted in three stages to ensure that different programmes were sampled. For example, the initial 10 interviews did not capture any peer-mentoring programmes or programmes provided by school staff.

Experts in the field were identified through the literature and via consultation with other researchers and then also purposefully selected and invited to take part in semi-structured interviews. The aim was to identify experts from both academic and practitioner backgrounds who could share their experiences of having run or worked with a range of different mentoring programmes and who could therefore help gain insight into broader contextual aspects of mentoring programmes.

All participants were contacted with an invitation letter or email, containing an information sheet explaining the research study. Invitations were followed up with a further letter, email or telephone call. Following participants' expressions of interest to take part, telephone interviews were arranged. There were no financial incentives to taking part. These interviews were collected as part of a wider study with the aim of developing a typology of mentoring programmes and findings from this have been reported elsewhere (Busse, Campbell, & Kipping, 2018).

### 2.2 Data collection

Telephone interviews were carried out by the main author of the study and facilitated using a semi-structured topic guide. The topic guide included open questions about the history, structure, organisation and characteristics of the mentoring programme, perceived challenges and opportunities and about any changes to the organisation or programme. Interviews were audio-recorded using an encrypted audio recorder (Olympus DS-3400). Interviews were conducted concurrent with data analysis in three waves between September 2015 and March 2017. Interviews lasted on average one hour.

Interview recordings were transcribed verbatim by the main author of the study and then anonymised. All data were stored on a secure, password protected, electronic database. Hard copy data such as reply slips and consent forms were held in locked filing cabinets at the University of Bristol, United Kingdom.

### 2.3 Analysis

Transcripts were read and re-read to allow familiarisation with the dataset and to begin the analytical process. Whilst doing so, initial thoughts and impressions were noted. Segments of text were summarised in the document margins and any thoughts about the data itself or occurring concepts were noted. Where necessary, certain parts of audio-recordings were listened back to in order to clarify meaning. In line with inductive analysis techniques, when summarising parts of texts, the participants' original wordings were used wherever possible (Charmaz and Belgrave, 2002). Data analysis began immediately after production of each transcript in the attempt to carry out data analysis iteratively and concurrently to data collection. Data were analysed thematically (Braun and Clarke, 2006). Each time a new code was identified, this was added to a list of codes, which was then adapted into a coding framework. Codes were created and compared using constant comparison derived from grounded theory (Charmaz and Belgrave, 2002). Based on the categories and codes derived from the data, an initial conceptual framework, or index, for the study was built. The framework was further refined and adapted as the data analysis progressed and further categories and themes emerged.

A selection of transcripts was shared with two other members of the team who were not involved in the data collection. Where there was disagreement, original interview data were scrutinised against the coding and discussed until agreement was reached. Data from all transcripts was labelled or indexed accordingly, by identifying relevant parts of text in transcripts and assigning them to one or more of the categories in the conceptual framework in NVivo10. All researchers agreed on the final emerging themes and the representations of such.

### 2.4 Ethical approval

Ethics approval for the study was granted by the Faculty of Medicine and Dentistry Research Ethics Committee (FREC) in 2015 (reference number 24341). All participants provided written consent to take part. Experts in this study were asked for their consent to be acknowledged by name as it would have been difficult to preserve their anonymity.

## 3 Results

### 3.1 Description of participants and programmes

Twenty-three of 29 programme managers approached (79% response rate) and all 5 experts in the field of mentoring took part in interviews. Managers worked in organisations such as secondary schools, universities, mentoring or youth service organisations and were involved in running a total of 28 diverse youth mentoring programmes. Programmes were run within schools, communities or online and these were located within one or more of the four nations of the UK, had been in existence from less than two to over 16 years and varied in the number of young people supported each year (from 25 to over 5000 youths per year). The majority of programmes were delivered face-to-face using adult mentors, whereas some programmes were provided online or used peer mentors. Programmes differed in the frequency and length of mentoring sessions, time of delivery and overall programme duration. Mentoring provision by secondary schools or universities were usually internally-funded whereas programmes delivered by mentoring organisations and youth organisations were externally-funded. A typology of programmes has been previously published by authors (Busse et al., 2018).

Experts taking part in interviews were from both academic and practitioner backgrounds and had worked in the field of mentoring for several years, in all but one case over ten years. Some were involved in delivering mentoring programmes themselves.

Participants spoke in detail about how their programmes were developed, delivered, maintained, and sustained and emphasised the range of challenges and opportunities that they perceived. Each of these areas will be discussed in turn. When speaking about their programmes, participants highlighted that these areas are informed by contextual factors and, as such, that wider contextual factors impact on the development, delivery and maintenance of programmes. Quotations from programme managers have the reference M and experts the reference E. Table 2 highlights the key challenges and opportunities experienced in the development, delivery and maintenance of mentoring programme.

**Table 2:** Key challenges and opportunities in the development, delivery and maintenance of mentoring programme.

	Key challenges and opportunities outlined by participants		Supporting quote(s)
Developing a mentoring programme	(i) generating the idea for a programme	• Noticed decline in youth support services, changes in family structures and fewer places for youth to meet gave rise to mentoring programmes in the UK • Mentoring as a flexible and individualised ‘amenable’ intervention • Support for mentoring from schools and businesses • Mangers' belief in that mentoring works	“We kind of got this idea of using these young [people] as positive young role models in the life of other young people who maybe had an absence of a role model. […] The youth mentoring kind of really came from that sort of process.” (M12)
	(ii) trying to gain support from others for the programme	• Gaining trust from those inside and outside of the organisation described as time-consuming and difficult • Internal school mentoring programmes had to gain the support from senior school staff, mentoring organisations or youth organisations had to gain trust from their partner organisations	“The main challenges were […] to gain the trust of the local professionals, so social worker, education, police […].” (M14)
	(iii) deciding upon a programme model	• Decision related to broader programme characteristics; e.g. type of mentoring programme offered, aims and objectives, referral criteria, voluntary nature and targeting criteria • Decision making was described as iterative, collaborative and time-consuming and was made in partnership • Decision making involved acquiring knowledge about circumstances, resources and preferences within the local context • For rural areas, where travel takes longer and where face-to-face meetings are difficult to arrange, online mentoring programmes were considered	“From the school's perspective it's always easier just to work with a whole year group or with the whole class […] partly due to timetabling and […] it's just sort of much easier to say, let's just go with our whole of year 12 rather than us picking out particular students.” (M20)
	(iv) obtaining the necessary funding to run the programme	• Previous or current government initiatives were used to obtain funding for programmes • Some organisations sought funding from corporate sponsors or through school's pupil premium funding • Trends in funding streams were described and mentoring programmes would align their programmes and aims to match these to obtain funding	“There are trends in […] funding streams […] people that are funding you, they have got their own aims, and obviously, you got to be very aware of what it is that they want to see you achieve. […]” (M10)
Delivering a mentoring programme	(i) setting up the programme	• Need to appropriately train volunteers to become mentors • Setting expectations often formed part of the introduction and initial mentor training and perceived as critical to prevent mentor drop-out and turnover • This also involved thinking about whether or not a waiting list of young people wishing to be on the programme was going to be kept	“If we have [university] students, we give them a real grilling in terms of […] ‘You are working with vulnerable young people, is this definitely something that you can do?’.” (M8)
	(ii) developing programme infrastructure and policies	• Programmes differed in the extent to which programme infrastructure and policies were developed • Overall structures concerned people and participant management (keeping track of referrals, contact information, etc), programme monitoring (sessions, engagement, progress, etc) and programme management (policies, documentation, etc) • Many participants mentioned using database systems and technology to aid the management	“I've got a set of work paperwork in, in place, […] I've got the contracts, I've got the letters home, […] I've got a whole system in place” (M15)
	(iii) risk management	• Range of strategies used to manage and lower potential risks, including specific recruitment procedures for mentors, undertaking risk assessments and developing monitoring systems for programmes, particularly regarding online mentoring programmes • The legal requirement to safeguard was emphasised and interviewees described risk management as being a potentially long and arduous process • One participant highlighted that safeguarding requirements typically state that no adult in a volunteering role should be alone with a child which does not align with mentoring programmes where that is the main modus operandi • Risk management also influenced contact guidelines for mentor and mentee outside of the mentoring session; some had strict practices around no contact outside of programme, others did not	“Concerns about safeguarding is one of the things that limits males from being involved in this.” (M8) “And mentoring projects are quite a unique thing. I remember when I came for an interview and when they asked me about safeguarding, and I said, ‘well, obviously one of the biggest safeguarding challenging is, we discourage the best practice not to work 1-to-1 with a child or any young person’. And then here is the project based on that very premise.” (M8) “The only communication that the mentor has with the young person is during the session, […] the main reason for that is that just boundaries […] and it's been very successful and it also takes the pressure off the mentor […] and actually, parents and carers are really appreciative of that.” (M14)
	(iv) working with external partners	• Need to clarify roles and responsibilities with partner organisations, sometimes involving a partnership contracts or service level agreements • Other individuals that were part of the delivery of programme were mentees, mentors, programme staff, school liaisons, individual teachers and mentee's parents or carers • Schools and partners were seen to have possible different agendas for why mentoring programmes were used • When working with mentors from workplaces, participants reflected that mentors had to be released from work, which often had the consequence that sessions took part less frequently	“So we have, we hold a service level agreement with the schools which details that they are responsible for matching, choosing pupils and matching our mentors to specific individuals.” (M1) “They [mentors] have to get time off work. So we don't want to ask […] their employers to release them from work […] we would expect them to see their students at least once per half term.” (M19)
	(v) working with schools	• Practical considerations in schools included lack of meeting space and facilities, needing security clearance for mentors and the organisation of the mentoring sessions • Some schools were not willing to release students, particularly older students, during class to attend the mentoring sessions • Managers alluded to the key role of having a school liaison person to help organise the sessions, remind students and contact mentors • Scheduling issues meant that most programmes were delivered before or after school or within lunchbreaks, meaning that young people had to be willing to engage with the programme (e.g. by making programme voluntary) – however this also meant that some young people could not take part due to transportation problems or after-school commitments	“You do get some sort of like strong head teachers with their agendas. And it is about, sort of communicating, and […] that conversation regarding sort of like the appropriateness of the pupils sometimes [to be on the programme…] now and again, there is a separate agenda going on in the school.” (M7)
	(vi) making the programme work for a specific locality	• A few participants mentioned piloting or trialling their programme before making this available to more young people • Despite the requirement to have a clear delivery model and structure, participants highlighted the need to be flexible in the delivery of a programme as to consider individual needs from mentees, partner organisations, schools and the given circumstances within a locality (e.g. more practical for a school to select a whole cohort for a programme rather than selecting individuals) • The need to be flexible extended from programme content to the overall duration of mentoring, frequency, intensity and timing of the individual mentoring sessions	“It is usually a couple of hours. […] it depends, so it could be 2 to 4 h, or it could be whole days.” (M9)
	(vii) managing the end of the mentoring relationship	• Different practices and views were shared about preparing mentor and mentee for the end of the mentoring relationship relationships • Some organisations stated having an open-end mentoring programme that can continue after the normal duration in a formal way, some allowed mentors and mentees to meet informally, others required an official end to mentoring • Some acknowledged being in the process of evaluating their exit strategy and considering this particular aspect of mentoring further • Some organisation mentioned reducing their time towards the end of the mentoring programme, a few organisations mentioned organising an awards or prize giving evening and formally celebrating the mentoring experience and having taken part in the programme or providing mentor and mentee with a certificate • One participant queried whether the mentoring relationship should end once the mentor and mentee had achieved the set goals or whether this should continue • Some completed specific exit forms as part of the procedures, or having a specific meeting, or ‘keeping in touch days’ • As part of the end, a few providers also mentioned reviewing the mentoring process and how this has previously led to changes made to the programme model	“At the end of the year of mentoring, we, do an exit strategy with the young person, with the mentor and with the parents and carers […], and we start winding down the mentoring say in the last four to six sessions, […] and moving into other support […] but if they still feel like they would like to be mentored for another year, and the mentor is happy to do that, then we begin again with another year.” (M14) “If the student is still in school and their mentor leaves, or even if the relationships comes to an end, we always leave it open ended. We always say to the students ‘if you feel that you need to talk to somebody again, come back and see us’.” (M19) “We ask our, we kind of ask our volunteers to stay ‘til the end of the year, obviously, we kind of prepare for the last week. We have a celebration and a-a price-giving. Things like that.” (M3) “And it's supposed to focus for us is that the volunteer and the young person shouldn't meet up after the match is over. And the reason for that is that young people need to learn that endings can be positive.” (M6)
Maintaining a mentoring programme	(i) Ensuring continued funding	• Participants gave examples of programmes that had ceased existing because of a lack of funding • Participants explained how their programme staff received redundancy notes on an annual basis, and how programmes had to deliver on a range of outcomes mid-term to secure their continued funding • Participants explained that much of their time was spent ensuring continued and additional funding and described that obtaining funding from multiple sources can act as a protective factor, in case one source of funding ceases • Some participants explained how initial funding allowed programmes to set up the relevant structures that were then maintained in the future and required less funding.	“We always look for additional funding, looking for more funding and I guess it always is a constraint, because if we had more funding, we would be able to reach more young people and hire more staff and that.” (M12)
	(ii) Management of partnerships	• Managers talked about a range of ways in which they accommodated the needs of the partner institutions, such as providing progress reports to partners.	“We would ensure that whoever would be referring them into the programme had information on the progress.” (M2)
	(iii) Engagement of mentees and mentors	• Engagement of mentees was described as difficult at times and it was described as challenging to keep young people interested in the programme • Reasons for not engaging in the first place were due to young people not liking the programme or the idea of having a mentor or moving away • Managers acknowledged that different types of mentoring programmes required different strategies to foster engagement (e.g. an initial face-to-face session, closely monitoring and reminding to engage was beneficial for online mentoring programmes) • Other strategies to foster engagement on part of the young person were: highlighting the voluntary role of the mentor, making the programme voluntary, allowing the young person to choose a mentor, and signing a contract at the start of the programme • Ensuring continuous engagement was also perceived necessary for mentors • Participants expressed finding it difficult if a mentor was found not to engage and one expert expressed that some mentors might feel overwhelmed by what they encounter in programmes • To prevent mentor drop-out, interviewees highlighted setting expectations, providing mentors with wanted training and references as helpful, providing a good volunteering experience overall • One participant alluded to the fact that it was seemingly becoming more common for volunteers (i.e. mentors) to change the charities or organisations that they support, making this aspect particularly pertinent	“There are initial issues with parents, collecting them in the evening, timetable clashes, you know, they don't like it, or they move to another area.” (M3) “I do remember people saying that there is huge problems with the recruitment, you know […] a mentor might turn up for one session, but not turning up the next. […] that may well be because mentors are overwhelmed by what they are faced with.” (E5) “I mean it's infuriating if a mentor decides not to engage with a programme because they have volunteered and you got a mentee that wants to engage with them and […] that can be disruptive and damaging for […] that young person.” (M13)
	(iv) Assessment and evaluation	• Programme assessment and evaluation helped participants to learn about the group of mentees involved in the programme, to highlight gaps or needs within the programme and to demonstrating the potential effects of their programmes • Mentoring often occurred alongside other forms of support and services which made it difficult to disaggregate the potential influence of mentoring from other interventions • Experts alluded to the difficulty this presents of: (i) the complicated nature of examining causal relationships in mentoring, (ii) outcomes being individual to young people, and (iii) good mentoring resting on the quality of human relationships • Many participants alluded to the difficulty of measuring outcomes, differences between funder and organisation's aims • Some participants were currently involved in undertaking assessments or evaluations	“Especially in the third sector and especially for charities […] monitoring and evaluating is always something that we struggle with a bit. Just because it's very hard sometimes to, you know, quantify what you are doing.” (M12) “And unfortunately, funders and government always want hard outcomes but when you are working in such a, a sort of personal area, it can be very, it can be very hard to translate the outcomes of the programme.” (M12) “People's problems aren't solved in isolation, you know, there is this idea that you have a social ecology around you [….] [mentoring] it's not a cold science. It can't be. It is a human based.” (E4) “Whether that student has got a C in Maths, you know, to us that is not as important. But obviously […] in a school environment, that's very important.” (M19) “We'd want to measure raised academic achievement but we have not really been able to do that so far. […] we have not really thought of a way yet […] that we can best do that.” (M12)
	(v) Adapting the programme for long-term sustainability	• To adapt the programme, some interviewees mentioned having processes in place such as regular or annual programme reviews and feedback evaluation meetings with partners or steering groups • For example, some participants spoke about how the length of the mentoring programme was reduced to prevent young people becoming dependent on a mentor • Programme changes also occurred based on increased knowledge about mentoring and insight of how mentoring evoked changes (e.g. some participants felt that a longer duration of mentoring helped building the mentoring relationship, and consequently led to better outcomes) • Programme structures and policies became more detailed and refined with increasing experience	“We did a consultation with young people […] And a lot of the young people had then said, ‘well, actually being matched for a year isn't very good for us’ because then at the end of the year, you take away this person that I met weekly and I am left then with nothing. So, we looked at this and we decided that we would shorten the match, so they now get like a 3–6 months” (M6) “To give the young person the opportunity to develop the relationship, we can't just sort of fit it all into half an hour […] It's got to be worth turning up for. Otherwise young people won't turn up.” (M10)

### 3.2 Development of mentoring programmes

Development of a mentoring programme involved four key aspects: (i) generating the idea for a programme, (ii) gaining support from others, (iii) deciding upon a programme model and (iv) obtaining the necessary funding to run the programme. Managers explained that programmes were established because of the perceived or actual need for certain young people to get extra support and guidance to help them cope with challenges. By providing young people with an older, more experienced role model, through a flexible and individualised ‘amenable’ intervention, participants felt that this need could be met. Experts noted a rise in the overall number of programmes available in the UK in last three decades and observed that mentoring programmes were now more embedded in schools. The support for mentoring programmes in the UK was seen to have arisen out of changing family structures and the decline of other sources of support for young people, such as youth and social work. Similarly, participants noted that there were fewer places for young people to meet. Programmes were also seen to provide an outlet for adults in the community who wanted to help young people who they saw struggling.

*“I suppose some of it was in settings where the adult mentors were striving for that kind of relationship […] they were trying to sort of be someone who could help a young person.” (Expert, E5)*

*“Most organisations deliver mentoring programmes to the need that they are looking for.” (Manager, M2)*.

Participants pointed out that, compared to statutory interventions, mentoring programmes offered a different, possibly ‘better’, approach to working with young people. This was because mentoring programmes were voluntary and young-person led. An underlying belief in mentoring as a beneficial and valuable intervention was expressed by many managers:

*“The key reason that we use mentoring is because, I*'*d say it*'*s a youth work approach, and it*'*s not a statutory based intervention so its voluntary […] that young people engage in the service” (M2)*.

Some managers drew upon the fact that young people desired mentoring themselves. They also highlighted the interest of schools in mentoring, explaining that it helped teachers deal with increasing pressures, that it relieved them from having to deal with difficult students and that programmes could provide individual support for students that would otherwise have been missed by the school.

Once the idea for a mentoring programme arose, support and trust both from within and outside the organisation had to be gained. Gaining trust was described as essential to the success of a programme, yet difficult to obtain and potentially time-consuming.

*“If you haven*'*t got the support of your senior team in the school, it*'*s [mentoring programme] gonna fail.” (M15)*.

Furthermore, organisations had to decide upon a type of mentoring programme which involved acquiring knowledge about the circumstances, available resources and preferences in the given locality. Moreover, a decision had to be made about the type of mentor, setting, management and procedure of referrals, referral criteria, voluntary nature of the programme and targeting criteria. Decisions about the type of programme were made in collaboration with local partners and undertaken in an iterative and collaborative fashion.

Finally, managers addressed the need to obtain funding for the programme which was described as a key challenge for many organisations. Interviewees acknowledged that funders had a key influence on the chosen programme model, approach and aim to mentoring, programme size and structures:

*“Our funders then kind of dictate the area and the types of young people that we work with.” (M6)*.

The support for mentoring by schools was shown in schools' willingness to pay for the mentoring, oftentimes by spending their ‘pupil premium’ funding (additional funding for students eligible for free school meals in English schools). Likewise, participants emphasised that businesses and universities had an interest in mentoring programmes to align with their corporate social responsibility and referred to the availability of government funding for mentoring programmes.

In sum, opportunities arose because of widespread support for programmes from a range of institutions, willingness of these institutions to fund and invest in programmes and overall support and an underlying belief in mentoring as valuable for young people.

### 3.3 Delivering a mentoring programme

Managers highlighted seven challenges and opportunities of programme delivery: (i) setting up the programme, (ii) developing programme infrastructure and policies, (iii) risk management, (iv) working with external partners, (v) working with schools, (vi) making the programme work for a specific locality and (vii) managing the end of the mentoring relationship.

Managers primarily talked about the necessity and challenge of recruiting mentees and mentors to take part in the programme. Whilst recruiting peers to act as mentors was not perceived as difficult, recruiting ‘good’ mentors in volunteer-based programmes was a challenge:

*“The number-one challenge is getting enough good volunteers […]” (M5)*.

Setting up a programme further included consideration of how the programme, people and participants would be managed and monitored. In most cases, organisations appointed programme staff, such as programme managers, to oversee the programme. Developing suitable guidelines and policies was regarded as critical to the delivery, yet time-consuming.

One key concern for all organisations was to ensure that risks of the programme to those involved were appropriately managed. This was a particularly pertinent issue as many programmes worked with vulnerable young people and were delivered on a one-to-one basis in sometimes isolated areas.

*“Putting everything into place for safeguarding young people, and our mentors, that*'*s a massive challenge.” (M10)*.

The initial mentor training typically covered safeguarding and specific instructions for mentors to pass on any concerns or issues to programme staff or teachers. Some managers reflected that this might involve breaking confidentiality, in other words, telling the mentee that something raised confidentially as part of mentoring had to be passed on. This was regarded as a particularly challenging aspect as programmes wanted young people to trust the programme and mentor.

*“If the [organisation] says you must disclose this kind of stuff where does that leave someone who feels, you know, where the young person has said, I am telling you, because I can*'*t tell anyone else?” (E5)*.

To mitigate risks within school-based programmes, interviewees spoke about having coordinators and other staff nearby to oversee the mentoring, and the ‘private but public’ nature of mentoring within the school context. Despite a few programmes being run with close monitoring structures, most programmes worked on a “managing by exception” rule which stipulated that mentors were expected to inform programme staff only in the case of child protection related issues or concerns:

*“We have a kind of ‘managing by exception’ rule, so they need to detail if there are any concerns they have got, ok, and […] they will all have a monthly review.” (M3)*.

All programmes except internally-run school-based programmes required partnership working. Partners were essential within the process of making the mentoring programme happen and could involve schools, social workers, health services, police and local authorities. Working in partnership took different formats: from an institution solely being involved as an organisation to refer young people or mentors into the programme to being responsible for the (partial) delivery of the programme.

Many managers spoke about their experiences of working with secondary schools. Practical considerations mentioned included lacking space and facilities within schools to undertake mentoring, needing security clearance for mentors to work within schools and having difficulty organising mentoring. Managers acknowledged that schools are pressured environments and that the school's overall focus is on learning, which took priority over mentoring and led to sudden changes or cancellations of mentoring sessions.

*“We have occasions where we are all set up to go into a school and then at the last minute the school suddenly just says, ‘sorry, we can*'*t do it, we got this happening’.” (M24)*.

Furthermore, one expert noted that some individuals, such as teachers, might not like mentors coming in to deliver mentoring:

*“And I think often teachers resent mentors coming in from outside because they are able to do things that they feel they can*'*t to.” (E5)*.

Managers revealed that some schools might have different agendas from mentoring organisations that would need to be negotiated. Some schools chose students despite them not meeting the referral criteria:

*“They [mentees] are definitely not the most dysfunctional, though, saying that, some of them are because the school is so desperate for some support for these children, they put them on the scheme.” (M22)*.

To make the programme work for a given locality, managers emphasised the importance of partnership working and being flexible in the delivery of a programme. This meant possibly considering altering the programme model and structures:

*“We do a bespoke package for each child […] what we noticed is that not one size fits all.” (M16)*.

Attention was also given to the end of the mentoring relationship, with different practices in different programmes including “phasing out” the mentoring, stopping mentoring altogether or allowing mentor and mentee to continue to meet (see Table 2).

### 3.4 Maintaining a mentoring programme

Five components to maintaining a mentoring programme were identified: (i) ensuring continued funding, (ii), managing partnerships, (iii) engaging mentees and mentors, (iv) assessment and evaluation and (v) adapting the programme for long-term sustainability.

Differences were observed with internally and externally-funded programmes. In general, these challenges were greater and more central to the experience of externally-funded compared to internally-funded programmes.

One key factor emphasised by many participants of externally-funded programmes was the need to continuously receive funding to maintain and sustain their programmes long-term. Participants referred to occasions when funding was changed, cut or withdrawn, which emphasised the insecurity faced with short-term funding.

*“Insecure funding is, it really does affect everything.” (M10)*.

Funding insecurity led to worries and concerns of those working in mentoring organisations. Whilst the challenge of funding was a recurrent theme, some benefits from funding uncertainty were given: the need for organisations to think critically about their programme and programme theory, to develop structures for evaluation, ultimately leading to more evidence-based practice and expanded knowledge about ‘what works’.

Accommodating the needs of external partners was another major factor in enabling successful programme delivery. Managers described actively managing external relationships, emphasised the importance of not overburdening their partners and referred to changes made in the programme to accommodate partners' needs.

Moreover, the need to engage mentees and mentors and manage their expectations was emphasised. Whereas peer-mentoring programmes within secondary schools were typically oversubscribed, providers of other programmes using volunteer adults spoke about difficulties in recruiting and retaining enough mentors. Strategies to foster continuation in mentoring and to manage expectations were also highlighted (see Table 2).

To maintain programmes long-term, most participants and all experts acknowledged the importance of assessing and evaluating programme outcomes. Interviewees emphasised that this was particularly of priority for externally-funded programmes.

*“We know that grant-funders now are more interested in evidence-based approaches so what we had to do was introduce a standardised evaluation framework” (M23)*.

However, in many cases evaluations were not a priority and only undertaken if problems arose. Outcomes and evaluation for programmes were regarded as a particularly pertinent issue in the sector and one that organisations struggled with:

*“If there is something wrong, then there*'*d be a bit of research or investigation.” (M10)*.

Participants acknowledged that despite a range of mentoring programmes in existence, the actual evidence on mentoring programmes was weak and potentially not in line with the impact that was expected, making the evaluation of programmes a difficult one.

*“There is very little research […] about the impact mentoring programmes have. It*'*s terribly popular, everyone loves it, but when they actually try to track hard impact, it*'*s always been a little bit disappointing.” (M5)*.

Participants spoke about what outcomes they measured, how, and that this differed widely between programmes. Outcomes measured ranged from proximal to distal and spanned a range of topics, including programme experiences, educational outcomes, and risk behaviour outcomes. Less interest seemed to be shown in measuring health outcomes. The type of outcomes measured were generally based upon the aim of the programme. Participants drew a distinction between what was referred to as ‘hard’ and ‘soft’ outcomes: hard ones being relatively direct and tangible to assess by looking at educational records, while soft ones referred to those outcomes which were more challenging to assess and often based on self-report such as changes in self-esteem or confidence. Methods for capturing outcomes included qualitative interviews, questionnaires and surveys, monitoring forms, school records or other available data.

Generally, outcome evaluation and assessment seemed to be an ‘area in progress’ as many providers described currently looking for ways in which they can best measure programme outcomes. Some participants acknowledged having limited experience in evaluation and were hesitant about what to measure and how.

Another part of programme maintenance included the adaptation of the programme and programme structure. All participants mentioned having undertaken programme changes over the years and this was regarded as inevitable to keep the programme relevant, acceptable and valuable for everyone involved.

*“We like to change that [mentoring programme] every year because we feel the community changes every year” (M16)*.

Participants gave insight into changes that were made in the past to their programme, including changes in referral criteria, referral organisations, setting, programme model, programme structures, size and staffing. The potential dependency of mentee and mentor was voiced as an issue by a handful of participants who explained that, to overcome this, they changed the frequencies at times, made programmes less frequent towards the end, and, in the case of one programme, having mentees swap mentors.

Most participants intended to expand their programmes, highlighting the necessity and importance given to ensuring the continued operation and sustainability of programmes.

*“Over the next kind of two years we are […] looking to roll the programme out to different regions.” (M12)*.

### 3.5 Contextual influences on programme development, delivery and maintenance

When speaking about the development, delivery and maintenance of programmes, programme managers and experts recognised that formal mentoring programmes operated within a challenging and complex field overall.

*“The field is a little more complicated than a straight forward thinking, is mentoring good or bad? It*'*s the context in which it is provided in.” (E4)*.

In their description of programme structures, it became apparent that formal mentoring programmes interacted with a range of contextual influences; factors that were external to the programme but that influenced how programmes were developed, delivered and maintained. Contextual factors were found to encompass various levels of influence:

(i) individual-level (i.g. mentee and mentor's own background)
(ii) interpersonal-level (i.e. parental support)
(iii) community-level (i.e. available resources and places to meet)
(iv) organisational–level (i.e. programme structures and regulations)
(v) policy-level (i.e. regulations, safeguarding)
(vi) societal factors (i.e. norms and perceptions of mentoring).

Taken together, these factors had significant bearing on how programmes were developed, delivered and maintained and, as such, the programme's overall sustainability. These influences were understood to be related and interlinked.

**Fig. 1:**
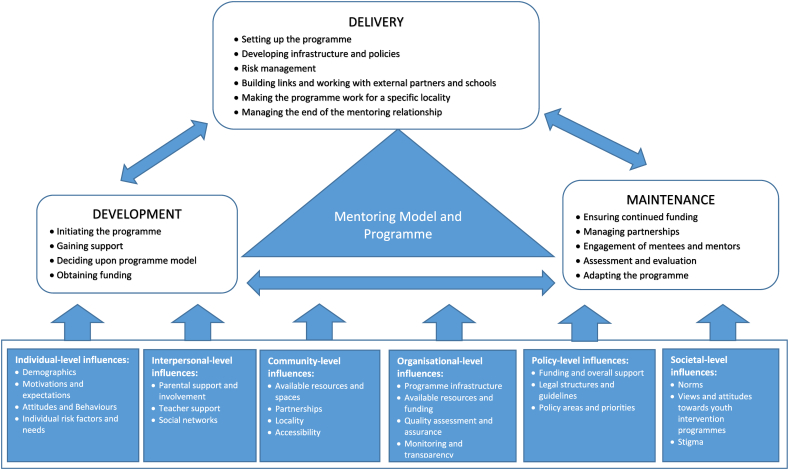
A conceptual model of contextual influences on formal mentoring programme development, delivery and maintenance.

Safeguarding requirements, a policy-level contextual factor, influenced the development of programmes by informing who was working with whom. For example, many programmes typically refrained from matching female mentees with male mentors and safeguarding policies were used to explain the lack of male mentors in many community-based programmes. Safeguarding requirements also influenced the delivery of programmes, for instance, as this was covered in the training with mentors, as this influenced programme infrastructure and monitoring and the setting in which the mentoring programme was provided. Finally, safeguarding policies also played a role with regard to the maintenance of programmes, as programme managers continuously assessed the risks of programmes. An overview of different influences specific to formal mentoring programmes is provided in Table 3 and these are summarised as part of a conceptual model in Fig. 1.

**Table 3:** Contextual influences on mentoring programme development, delivery and maintenance identified.

Levels of influence	Influences	Supporting quotes
(i) Individual-level influences	• Mentor and mentee backgrounds and characteristics • Motivations and expectations, attitudes and existing (health) behaviours • Individual risk factors of mentees	“They [selected mentees] would be on free school meals, or be identified as pupil premium students, so they would be identified by government, they would probably be first in family ever to consider university […] We would include all young people who are in care […] and young people with disability. Mental or physical, you know. So those are our criteria. (M24) “But if you got a white middle-class rower going into an inner-city London school, with high levels of deprivation, those children just can't relate to that person. The rapport is not built. […] So having somebody who lives around the corner from those children […] they have a much greater affinity with the children […] that local angle can't be underestimated.” (M23) “I also understand the need for some people to, to be paid, in order to be able to afford the time, it's very time-consuming […] but I then worry about it b*e*coming an obligation, as opposed to […] a commitment that you make to a young person” (E3)
(ii) Interpersonal-level influences	• Parental support and involvement in mentoring programme • Teacher and school support and cooperation • Available support from others • Mentee and mentor home circumstances • Mentee's social networks	“The actual children, attitudes, expectations, the parents are different. You know five, six years ago, most of the parents were on board, you know, they were, yeah, ‘ok, you are in charge, you know’ […] But today, it's more, they [parents] don't work with us as much, the more legal side of thing, ‘you can't do this’, you know, ‘legally, you can't do this, legally, you can't do that’.” (M16) “The parents in [name of mentoring organisation] generally are open to meetings with professionals and at least finding out what they got to say. […] But we, in [name of region in UK], get a real resistance from families. Less so from the young people, but a big resistance from the families.” (M17)
(iii) Community-level influences	• Available resources and spaces • Accessibility • Partnership working habits, history and practices • Other available services and interventions for young people within community • Connectivity and transportation • School resources	“I think, at that time, the UK and most of Europe was kind of different in terms of, family structures and other approaches. I mean there was no youth work in the States […] whereas mentoring took place within a context in the UK where there was […] a very strong youth work culture” (E5) “It's a very unique community […] it's very different to a city or a town […] everybody has got their kind of own agenda, people are very wary of new projects, it, they recognise a need in the community, it basically took us a good eighteen month get basically the professionals and parents to trust that our project was gonna be a professional, professionally-run project.” (M14) “I mean, in cities, there are many organisations that pull together […] big companies who have come in to social responsibility programmes, and so you get a lot more industry mentoring, when you are out here in the rural and coastal, you don't have much industry to draw upon for that kind of support.” (M24)
(iv) Organisational-level influences	• Available programme structures; including those around recruitment, matching, monitoring and supervising mentoring relationship • Available resources, including technology, documentation and infrastructure • Organisational history and past experiences • Linkage to other organisations • Staffing for mentoring programme • Agendas of partnership organisations	“It [mentoring programme] works brilliantly because it's been going for so long, it's almost automatic. It's like, we know what to do, you know.” (M15) “However, we won't accept self-referrals. We would be absolutely inundated. “(M10) “We offer a kind of monthly budget for the 12–21 year old programme […] pairs have that much to spend and there is some good life math in there for young people and their mentor to work through together.” (M8) “If you were looking at it from the outside and being very cynical, that universities have to meet certain targets [….] what they are doing mentoring for is to ensure that they have a […] supply of good students who will do the course. […] It is just, thinking about the kind of the external agendas. So is that a recruitment drive or is that about the relationships with the young person?” (E5)
(v) Policy-level influences	• Available funding for mentoring programmes • Legal structures and guidelines of concern to mentoring programmes, including safeguarding requirements • Policy areas and priorities	“And in the States [USA], it's for example, there were just too many legal implications to have individual mentoring where the young person, and the adults meet outside of school, so we can only do individual mentoring within the school setting.” (E3) “Quite a few of [different countries] run exactly the same […] mentoring programme but the target groups can differ.” (E3) “It was widespread that charities were founded by their local council […] And then with the election and the new government, it, you know, the funding for lots of charities was taken away.” (M22)
(vi) Societal-level influences	• Support for youth prevention and intervention programmes • Norms around mentoring programme practices • Cultural norms around mentoring programmes, evaluation of services • Possible stigmatisation of those taking part	“Some people saw it [mentoring programme] as a very negative thing, some people saw it as a very positive thing, […] two and half year down the line, we have got quite a few success stories its going very well now.” (M14) “Being in a small community, there are not too many places to hide. Whereas with a city, you know, you have got plenty of places you can go […] here, somebody is always gonna be saying, ‘Oh, I wonder what they are doing together?’” (M14)

Due to the challenging context within which programmes were developed, delivered and maintained, participants raised concerns that the challenges experienced could impact on the effectiveness of programmes. More specifically, experts were concerned about the possibility of mentoring programmes to do harm:

*“They are aiming to tackle long-term issues but […] their funding is precarious, they*'*re embeddedness in, you know, the life of the young people is very uncertain, […] one of the things that we were concerned about was the capacity of mentoring programmes to do more harm than good.” (E5)*.

*“I think there is pressure to sell programmes, […] and make matches at all costs. […] if a young person is having difficulty in their life and it*'*s decided that a mentor would be good for this young person, and you form a match and […] then for whatever reason the mentor packs it in […] closing that match can be very difficult. You know? So we need to be aware of the dangers.” (E4)*.

Potential harms alluded to were the fact that taking part in a mentoring programme might impact on other circumstances in the life of the young person, such as undermine their relationship with other adults in their life or their access to other services.

*“Can mentoring undermine those sort of relationships? What I mean by that is […] by the introduction of a child to a mentor […] are you ruling out the possibility that an aunt or an uncle could provide that support if they were encouraged?” (E4)*.

*“And the question that we have is, if that mentor had stepped away, would that [better living situation] have happened sooner? Or equally […] would it not have happened and that person would be in an even greater, more vulnerable situation? So questions without answers.” (M8)*.

In fact, some managers acknowledged that programme models or structures were not followed at times because of contextual constraints.

*“I mean to a certain extent we try to match them. But in reality, it comes down to timetabling and who is available.” (M7)*.

*“We ask the students if they prefer male or female because we want them to feel comfortable, and […] we accommodate that as much as we possibly can” (M22)*.

Experts raised concerns about the fact that organisations often had a vested interest in the delivery of programmes for the organisation's benefit and emphasised the need to look at external agendas.

One expert, in particular, highlighted contradictions with regard to the field of mentoring. One contradiction was the frequently short duration and short-term funding of programmes compared to the long-term outcomes programmes aimed to achieve. Another contradiction was programmes wishing to help young people but removing access to mentoring if a young person did not attend and by programmes typically recruiting successful mentors to work with what were regarded as ‘unsuccessful’ mentees.

*“There might be sanctions if the young person didn*'*t behave in an appropriate way, then the mentoring would be taken away from them.* “*(E5)*.

Some participants expressed frustrations with some of these contextual factors and external requirements and particularly drew attention to the insecurity of funding available for programmes. Securing the sustainability of a mentoring programmes seemed to be a ‘juggling act’ for many providers; having to cope with pressures and accommodating wishes and interests of partner institutions, having to engage mentees and mentors, managing expectations, and needing to keep partner organisations happy. This required organisations to be flexible in their approach and to be continuously evaluating programmes structures in order to keep the programme relevant, acceptable and valuable for all stakeholders involved.

## 4 Discussion

Formal mentoring programmes for young people in secondary schools in the UK are faced with a range of challenges and contextual influences in their development, delivery and maintenance. Key challenges and opportunities included funding, managing risks and expectations, partnership working, engaging mentees, mentors and stakeholders with the programme and adapting the programme to ensure long-term sustainability. Given the range of challenges experienced, securing the sustainability of a mentoring programme seemed to be a ‘juggling act’ for many mentoring providers. This study identified a range of interactive influences, occurring at the individual, interpersonal-, organisational-, community-, policy and cultural-level, which are summarised in a model. This highlights how mentoring programmes operate within a specific local and national context.

Differences were found between internally- and externally-funded programmes. Those running externally-funded programmes experienced the challenges to a higher degree, particularly concerning the funding and consequent need to assess and evaluate their programmes. Many of such programmes found themselves in a position whereby they could be seen to ‘hang by a thread’. This comparison between internally- and externally-funded programmes has not been of focus in the literature and deserves further exploration.

The current model of youth mentoring, which is used as a guiding conceptual framework for programmes by Rhodes (Rhodes, 2005; Rhodes et al., 2006), acknowledges the influence of the family and community on programmes. This work is seen to complement Rhodes' model and to expand our thinking about mentoring programmes by also taking into account organisational-, policy- and cultural-level factors. Similar proximal and distal influences have been described and theorised to impact on human and organisational behaviour, for instance, as noted by the socio-ecological model of health (Dahlgren and Whitehead, 1993). This is based upon Bronfenbrenner's theory of human development which distinguished between different systems, including a micro- and macro-system, which have an impact on individuals development and behaviour (Bronfenbrenner, 1979, Bronfenbrenner, 1986). Moreover, this finding relates to organisational management literature, in which organisations and programmes are conceptualised as working within specific contexts (Johns, 2006). These contexts, involving local and national influences, it has been argued, have an impact on organisational practices (Liu et al., 2015), performance (O'Toole Jr and Meier, 2014), and leadership appraisals (Hernandez et al., 2016). To our knowledge, this is the first time that explicit examples of different levels of influences have been given and identified as impacting on mentoring programme practices.

We reported that there are circumstances in which potential harm can arise in mentoring programmes. Specific areas that were highlighted were the end of the mentoring relationship and mentor characteristics, which are in line with the previous literature (Spencer, 2007). Due to the constant need to change, adapt and optimise their programmes and processes, we found that some programmes deviated from their original model, making the programme logic model or theory of change unclear. This has consequences for programme evaluation, making it difficult to evaluate programmes and finding out what works, how and why (Bonell et al., 2006; Hawe et al., 2004). Programme managers did not talk to a great extent about parental involvement within programmes, and although this might be less applicable in school-based programmes, this has been linked to the success of a programme (Pryce et al., 2015; Spencer, 2007).

Some contradictions within mentoring were described, highlighting the complex and possibly conflicting needs of programmes and participants. Programme managers spoke less about these potential conflicts and generally spoke in positive terms about their own mentoring programmes. Matching often unrelated and similar mentors and mentees has been previously alluded to in studies of successful mentoring relationships, where, for instance cultural awareness and understanding have been noted as key to establishing a trusting mentoring relationship (Spencer, 2006; Spencer et al., 2016).

Some of the named challenges such as the need to manage expectations have been discussed in the literature or past evaluation studies (Laco and Johnson, 2017; Spencer, 2007). Country-specific challenges were also reported, such as the need to safeguard and to break confidentiality when the safety of the participant or others is judged to be in danger, which is required by UK legislation. This might indicate that programmes are not necessarily transferable from one setting to another (Philip, 2003) and that care must be taken when adapting programmes from other settings (Brady and Curtin, 2012).

### 4.1 Strength and limitations

To our knowledge, this is the first time that programme managers and experts themselves have explicitly given voice to their experiences and the challenges and opportunities faced in the development, delivery and maintenance of mentoring programmes. This work is based on the participants' first-hand accounts of running mentoring programmes and the experiences of experts in the field.

The generalisability and representativeness of our findings with regard to countries outside the UK is uncertain. This is particularly the case as our study identified that specific contextual factors impact on the development, delivery and maintenance of programmes. Whereas the research aimed to provide a general overview, since each programme is operating within a specific setting and context, other mentoring organisations might face different or additional challenges not mentioned. Due to the sampling framework for our study, the experiences of smaller programmes or programmes without a web-presence were not included and their experiences might differ. The application of this research to other types of mentoring programmes, such as those working with adults, or primary school children, or in other countries, may be limited for similar reasons. The conceptual model (Fig. 1) has not been consulted upon with others and it represents a first attempt to address the specific contextual influences of mentoring programmes.

### 4.2 Research implications and future research needs

Mentoring programmes are situated within a specific and highly complex context. This study underlines the importance of taking into account contextual factors within programme descriptions and evaluations and for contextual factors to be captured as part of programme evaluations. For these reasons, this research suggests that mentoring programmes might work in one, but not necessarily in another context, meaning that findings from evaluations in the USA might not be easily transferable to the UK context.

Our study carries implications for mentoring researchers and practitioners in the field internationally as well as in the UK, the latter in which a recent rise of youth mentoring programmes has been noted (Children's Commissioner, 2018). In order for programmes to function effectively, it is valuable for programme managers to actively consider the context within which their programmes are funded and delivered and to attend to each of the areas raised concerning the development, delivery and importantly, the maintenance of the programmes.

Service managers need to provide a clear description of the programme logic and work towards evidence-based programme structures and characteristics. Whereas many programmes seemed to follow what is regarded as ‘good practice’, such as allowing the relationship to develop over time (Grossman and Rhodes, 2002) and comprehensive pre-match training and ongoing training (Rhodes et al., 2009), other programmes did not. This might impact on the programme's effectiveness and potential to do harm. It is unclear whether and in what ways the existing research-evidence based best practice criteria, such as the “Elements of effective practice” (Garringer et al., 2015), hold for UK programmes.

As some managers expressed a lack of knowledge regarding their outcome assessment, this might be one area for collaboration between practitioners and academics. Academics need to acknowledge the complexity surrounding programmes and recognise that programme models and structures may change over time. Service commissioners should closely consider programme factors and contexts and commit secure funding to evidence-based, cost-effective programmes.

Future research is needed to explore how contextual factors can impact on programme effectiveness to identify what works, for whom and under which circumstances. Given the lack of evidence of effectiveness of mentoring programmes in the UK, there is a need for a future effectiveness trial. We recommend that this includes the use of a cost-effectiveness and process evaluation to capture the contextual factors within which mentoring is provided. It would further be of interest to explore which programme-level moderators of effectiveness and organisational practices and contexts which lead to more effective programmes.

Research is needed to extend the present investigation and examine how the perceived challenges and opportunities relate to others involved in mentoring programmes, such as mentor/mentee, parents/carers or other stakeholders. Future research is also required to test the generalisability of the conceptual model presented both within the UK and internationally and to further extend the model to identify additional contextual factors not captured by it.

This study found that mentoring programmes undergo processes of adaptation and changes over the years, thereby changing what is delivered. Future research is required to explore reasons for programme adaptation and the extent to which this is documented and consequent implications for the theoretical stance of programmes and logic models.

## 5 Conclusions

This study highlights that the development, delivery and maintenance of formal mentoring programmes to operate within a complex context. This study identified a range of interactive influences, occurring at the individual, interpersonal-, organisational-, community-, policy and cultural-level. Findings from this study will help service providers, commissioners and academics think about the contextual influences on mentoring programmes to ensure that programmes are delivered as intended, sustained long-term and evaluated appropriately.

## Funding

The work was undertaken with the support of The Centre for the Development and Evaluation of Complex Interventions for Public Health Improvement (DECIPHer), a UKCRC Public Health Research Centre of Excellence. Joint funding (MR/KO232331/1) from the British Heart Foundation, Cancer Research UK, Economic and Social Research Council, Medical Research Council, the Welsh Government and the Wellcome Trust, under the auspices of the UK Clinical Research Collaboration, is gratefully acknowledged.

## Conflicts of interest

Declarations of interest: none.
